# On the Usage of GPUs for Efficient Motion Estimation in Medical Image Sequences

**DOI:** 10.1155/2011/137604

**Published:** 2011-08-18

**Authors:** Jeyarajan Thiyagalingam, Daniel Goodman, Julia A. Schnabel, Anne Trefethen, Vicente Grau

**Affiliations:** ^1^Oxford e-Research Centre, University of Oxford, Oxford OX1 3QG, UK; ^2^Institute for the Future of Computing, Oxford Martin School, University of Oxford, Oxford OX1 3BD, UK; ^3^School of Computer Science, The University of Manchester, Manchester M13 9PL, UK; ^4^Institute of Biomedical Engineering, Department of Engineering Science, University of Oxford, Oxford OX3 7DQ, UK

## Abstract

Images are ubiquitous in biomedical applications from basic research to clinical practice. With the rapid increase in resolution, dimensionality of the images and the need for real-time performance in many applications, computational requirements demand proper exploitation of multicore architectures. Towards this, GPU-specific implementations of image analysis algorithms are particularly promising. In this paper, we investigate the mapping of an enhanced motion estimation algorithm to novel GPU-specific architectures, the resulting challenges and benefits therein. Using a database of three-dimensional image sequences, we show that the mapping leads to substantial performance gains, up to a factor of 60, and can provide near-real-time experience. We also show how architectural peculiarities of these devices can be best exploited in the benefit of algorithms, most specifically for addressing the challenges related to their access patterns and different memory configurations. Finally, we evaluate the performance of the algorithm on three different GPU architectures and perform a comprehensive analysis of the results.

## 1. Introduction

Motion estimation is one of the fundamental and crucial operations in machine vision and in video-processing applications. The process is often computationally intensive, and minimising the time for estimation across a number of frames is often a key objective in interactive image/video processing applications. As we will see in forthcoming sections, the task is repetitive and renders itself for exploitation in parallel architectures. With the rise of multicore machinery, such as many-core microprocessors and graphics processing units (GPUs), it is natural that the abundant amount of parallelism available on these systems to be exploited by mapping the algorithms on them. Among these, usage of GPUs has become increasingly common across many scientific domains.

There are several reasons for such a wide adoption of GPUs across many scientific disciplines. Modern GPUs contain hundreds of computational cores and have become available at a fraction of the cost of an equivalent conventional CPU-based system. This relative measure of performance versus price and performance versus power ratios between GPU-based architectures and CPU-based architectures further encourages the choice of GPUs.

However, the performance gains are not without significant challenges. Firstly, the identification and exploitation of any parallelism in the application is the responsibility of the developers. Often, this requires extensive remapping work rather than simple program transformations and often change in the fundamental algorithm. Secondly, the GPU programming model is not oblivious to the underlying architecture. Detailed knowledge of the architecture is fundamental for writing effective GPU-based applications.

These issues are partly overcome by different programming models, such as OpenCL [1] or CUDA [2, 3]. In practice, although these programming models simplify the task of programming these devices, they are far from providing abstractions at the domain-specific level.

GPU implementations of some specific image processing algorithms have already been made available, including optical flow algorithms as those outlined by Marzat et al. [4]. However, in this paper, we consider a more complex, complete and enhanced version of the original optical flow algorithm. The motion estimation algorithm we use in this paper combines local and global optimisations and preserves the volume during motion estimation—a key requirement for cardiovascular medical image analysis. We then map our motion estimation algorithms on to three different GPU systems with appropriate optimizations. We have chosen the systems whose GPU architectures are representative of the time line and relevant to the important architectural aspects of GPUs. We effectively demonstrate the applicability of the algorithm using a set of three-dimensional image sequences. Using this as an evaluation phase, we discuss and highlight the relative merits and demerits of architecture-based realization of the algorithm and resulting impacts on the overall performance. To the best of our knowledge, in the context of GPUs, there is no comprehensive discussion of a motion estimation algorithm of this level in the literature. Our comprehensive analysis on the effect of architecture and programming decisions can be abstracted for different image processing applications. We believe this would be a highly valuable resource for (biomedical) image analysis researchers, and this is, thus, the fundamental aim and contribution of this paper.

The rest of this paper is organized as follows: Sections 2 and 3 serve as a background for the rest of the paper. We first discuss the GPU-based systems in Section 2, highlighting the differences to the conventional CPU-based system wherever applicable. Then, we discuss the mathematics behind motion estimation in Section 3 and concisely formulate the motion estimation algorithm. This is then followed by Section 4, where we discuss the implementation and mapping aspects in detail highlighting the architectural aspects wherever necessary. We evaluate the performance of the enhanced algorithm in Section 5 along with the presentation of our analysis. Finally, we conclude the paper in Section 6 summarizing our key findings and directions for further research.

## 2. Parallelism with GPUs

Exploiting graphics cards or accelerator cards for their computational capability is not a new concept. However, historically they have been exceptionally hard to program and demanded programmers to have a rather in-depth understanding of the cards, their instruction set, or familiarity with OpenGL calls and Shader languages. However, with the introduction of compute unified device architecture (CUDA) by Nvidia, this setting has improved rather significantly. The CUDA is both a programming model as well as a hardware model coupled together to provide considerably high-level utilization of the GPUs. As will be observed, it is still the case that an intimate knowledge must be maintained to leverage their potential, but it is relatively easier than Shader languages or OpenGL calls.

### 2.1. GPU Architecture

**Figure 1:**
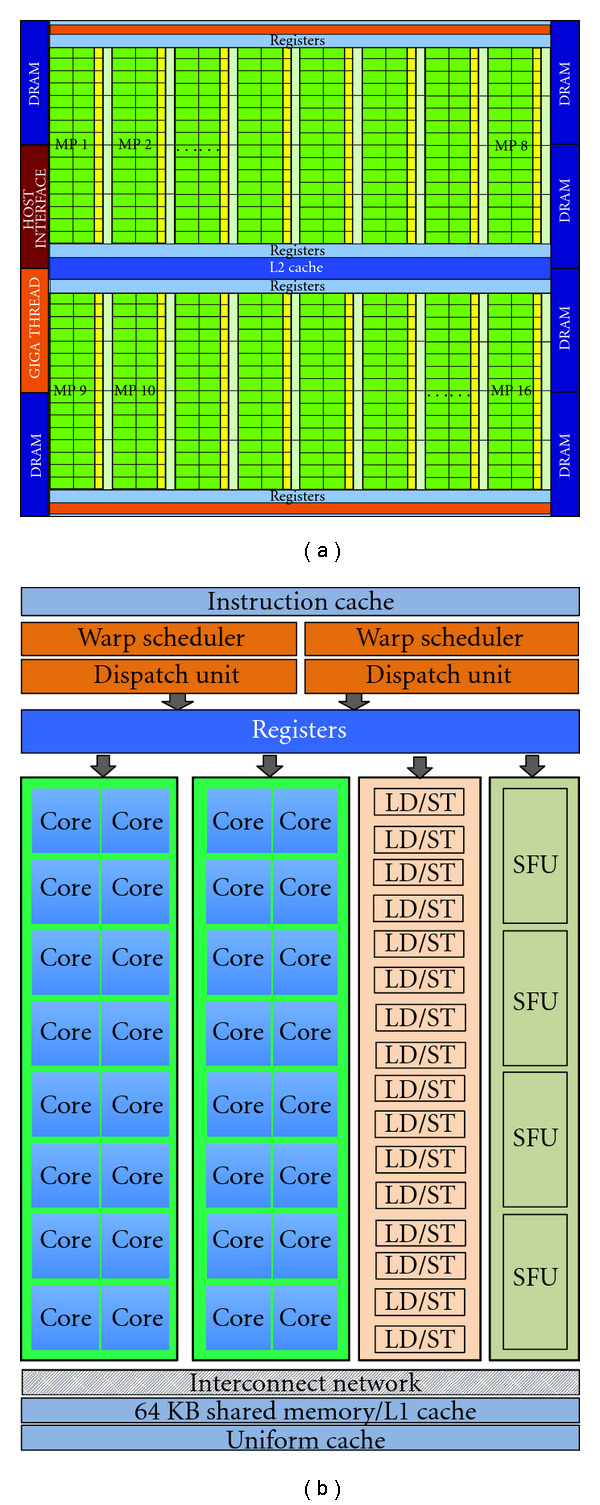
The overall architecture of a modern Fermi-based GPU device (a) and the inner details of a multiprocessor (b). Multiprocessors are configured around a shared level-2 cache and register files. Each multiprocessor has a number of computational cores and a level-1 cache. In the earlier versions of architectures such as C1060, these two cache levels do not exist, and the absence is facilitated by explicitly managed memories. This includes shared, constant, and texture memories. Image adopted from CUDA Programming Guide [2].

A compute unified device architecture- (CUDA-) enabled GPU is connected to the host system via a high-speed shared bus, such as PCI Express. We show an internal arrangement of a typical GPU in Figure 1(a). Each GPU consists of an array of streaming multiprocessors. Each streaming multiprocessor is packed with a number of scalar processing cores, named streaming processors, or simply cores. This is shown in Figure 1(b). These scalar processors are the fundamental computing units which execute CUDA threads. For example, the Nvidia Tesla C2070 GPU has 14 streaming multiprocessors and each streaming multiprocessor consists of 32 streaming processors, yielding 448 processing cores in total. The number of cores per multiprocessor or the number of multiprocessors per GPU varies from device to device. In CUDA, all threads are created and managed by the hardware. As a result, the overheads are almost negligible, and this leads to the possibility of executing a large number of threads at a time and to switch between them almost instantaneously.

Unlike multicore CPUs where the processor currently contains a relatively small number of cores each of which is capable of operating completely independently of each other, computational cores inside GPUs work in tandem and in a lock-stepped fashion.

Apart from the number of computational cores, one of the important aspects on which GPUs vary from CPUs is their memory subsystem. In GPUs, the traditional control logic dedicated to data management is used for computational cores, maximizing the space for these. This renders the data placement operations to be defined by the programmer with little or no assistance from hardware. However, to facilitate better placement strategies, GPUs are equipped with different memories. For example, in conventional CPUs, data placement is voluntarily done and in the absence of any placement, the control logic is responsible for raising the data through various different memory levels, to maintain coherency and to store them. In contrast, in the context of GPUs, the programmer is responsible for moving the data to appropriate memory. Such a liberated approach leads to considerably intricate programming model. For example, the way thread contexts are handled or how the data are moved around or the guarantee on the availability of the data prior to a computation are now left to the programmer.

Recently, GPUs have evolved rather significantly in this respect. GPUs are differentiated by their compute capability. The compute capability describes the features supported by a CUDA hardware. These features vary between devices from generation to generation in respect of maximum number of threads, support for IEEE-compliant double precision and alike. GPUs with a compute capability of less than 2.0, do not support any automatic data placement or coherency mechanisms. However, from devices with compute capability of 2.0, known as Fermi-based architectures, this has changed. Fermi-based devices contain cache memories but with the possibility of performing volunteer data management. This means that even in the presence of cache memories (see below), there is still some aspect regarding the data placement and management left to the developer. A more detailed information can be found in the CUDA Programming Guide [2]. 

In the latest generation of GPUs (based on Fermi architecture), the memory system is partially arranged in hierarchical manner and computational units are arranged alongside this memory system. A GPU typically has the following memory subsystems: global memory, a common level-2 cache, a combined private level-1 and shared memory, constant cache and texture cache. The global memory (also known as device memory) is common to processors (and thus to all threads) and has a high access latency. The private level-1 cache is exclusive to a streaming multiprocessor and has a low-latency connection. The level-2 cache is common across processors, and it has a better latency than global memory.

Both constant and texture memories are read-only memories separated from the shared memory. With the introduction of cache memories in GPUs, aggressive exploitation of both constant and texture memories is performed only when absolutely necessary. However, their load granularity (number of words loaded upon a load instruction) is different. As a result, sometimes, it is beneficial to utilize them. The constant memory allows each group of processors to store a selection of constants that they are going to use for the computation locally to allow fast access, without any coalesced memory access issues between processors. The texture memory provides read only access to data and follows a similar architecture to constant memory, except that instead of having a designated memory for the card as a whole, textures are bound to data stored in the card's global memory which leads to larger data capacity. As the memory is designed for storing textures in graphics applications, the memory supports a range of hardware-based functions such as interpolating the value of points that are not on integer locations.

Furthermore, the private level-1 cache is reconfigurable. This finite amount of memory pool can be configured so that part of it can be used as a shared memory, while the remaining is used as a cache memory. This enables the applications to receive partial data placement support. The system supports fixed number of such configurations, and a configuration suitable for a given application is often not known in advance and thus may need to be determined by experimentation. The total memory available for shared-memory and/or level-1 cache on current Fermi-based systems is 64 KB. This is normally used to buffer inputs and outputs to allow computations that do not naturally fit the coalesced memory access pattern to take advantage of the fast data transfers. This private first-level caches/shared memory are available to every streaming multiprocessor. In addition to this, there is a 768 KB shared secondary-level cache, which can be turned off if needed.

On GPUs, memory bandwidth to the computational cores is typically higher than that found on a CPU, meaning that cores are less likely to suffer from starvation for data. Furthermore, this connection often has additional optimizations if certain patterns of access are adhered to and often take the form of coalesced memory accesses. If the memory is accessed by threads at random (uncoalesced), each memory load is performed independently; however, if all the cores in a group in order access consecutive memory locations (coalesced), starting from an offset into memory that is a multiple of 16, then 16 memory loads can be done in the time usually required to perform a single one.

### 2.2. CUDA/GPU Programming Model

The CUDA programming model, which is an extension of the C programming language, relies on this hardware support to provide concurrency. In the model, computations are expressed as special functions known as * kernels*. A kernel is launched from the host-CPU and executed by *N* threads using the available computational cores (and *N* is usually in the range of several thousands) on the GPU. All threads are organized as a one- or two-dimensional grid of thread blocks. Each block can be one-, two-, or three-dimensional. Threads in a block are assigned to the same streaming multiprocessor during execution. With a unique numbering scheme for threads, each thread can be made to compute on a different subset of the input data so that the execution leads to the single program multiple data (SIMD) style parallelism. The memory system arrangement is such that potential data locality among threads can be exploited by computational cores.

The CUDA programming model evolved over time and originally the model relied on manual placement of data—which means that the application developer is solely responsible for moving the data from the host memory to the device memory (or in reverse direction) and to exploit any reuse by relying on shared memory or constant cache. However, modern GPUs partially support automatic data placement, most specifically to cache memories. As discussed in the previous section, the level-1 cache memory can be configured as cache memory or as shared memory or as both. Yet, it is the responsibility of the programmer to make the right judgement on the amount of memory to be dedicated for cache or for shared memory and to ensure that the latencies are hidden and memory requests to the device memory are linearized for best bandwidth exploitation (hardware memory coalescing). The hardware support is available only for the data movement from and to the cache memory. In line with the conventional parallel programming models, memory transfers (corresponding to communication overheads) may offset the benefits of parallelization, if it dominates the execution time. As a result, it is performance critical that memory transfers around the system and within the GPU are minimized as much as possible. For example, if a kernel feeds another kernel with its output, it is beneficial to retain the data in the GPU device memory without any intermediate transfers to the host.

The latest generation of CUDA devices support a number of other features which we do not explore in this paper. This includes the ability to launch multiple kernels and the utilization of unified memory.

## 3. Motion Estimation

### 3.1. Background

Images are fundamental in a wide range of biomedical applications, covering most aspects of medical research and clinical practice. Improvements in technology have brought increased resolution and higher dimensionality datasets (three-dimensional and higher); furthermore, studies involving several modalities are becoming more common. In these circumstances, it is indispensable to have means for automated image analysis.

Given the dataset sizes and the limited time per patient available in clinical settings, the speed of image analysis algorithms is crucial.

Imaging technologies have become integral part of all aspects of clinical patient management, from diagnosis to guidance of minimally invasive surgical interventions. Estimation of organ motion is necessary in many of these applications, either because motion provides an indication of the presence of pathologies (as in the case of cardiac imaging), or because the presence of motion is detrimental to the accuracy of the result (as in the effect of respiratory motion in the assessment of other organs).

**Figure 2:**
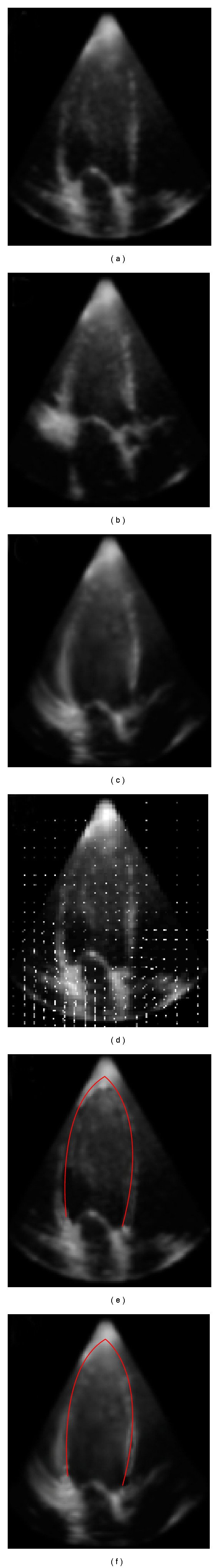
Example of motion estimation in a cardiac ultrasound sequence. (a) Original slice at end diastole. (b) Original slice at end systole. (c) Image (b) after alignment to image (a). (d) Estimated motion vector field, shown superimposed on (a). (e), (f) Images (a) and (c), respectively, with the endocardial contour superimposed. Note how the shapes are matched by the motion estimation process and all motion estimation and resampling were performed in 3D; a sample 2D slice is shown for clarity.

Motion estimation has been profusely investigated in machine vision and video coding applications, where minimising the time for estimation across a number of frames is often a key objective in interactive applications. Medical imaging shares some (but not all) aspects with these and adds the common use of three (or higher) dimensional sequences. As an example, so far widely used two-dimensional echocardiography (ultrasound imaging of the heart) is being gradually replaced by real-time 3D echocardiography (RT3D). RT3D scans can typically consist of 200^3^ voxels per frame, with approximately 20 frames per scan. Local estimation of structure motion is required for assessing a range of heart conditions. In order to be fitted in the clinical protocol, this estimation would need to be done in a few seconds, ideally in real time. In Figure 2, motion estimation is illustrated in a sample echocardiographic sequence. Many other applications within the biomedical imaging field exist, in some routine clinical cases reaching image sizes of 512^3^ voxels per frame, which can be much larger in basic science applications (e.g., analysis of histopathology slices).

A number of algorithms have been proposed to estimate motion in medical image sequences. In fact, the problem of motion estimation is sometimes just considered as an image registration (alignment) procedure, where registration between consecutive frames, or between each frame and a specific one selected as reference, is performed. This opens up the possibility of using any of the approaches proposed in the extensive registration literature. For an overview of registration methods, the reader is addressed to reviews such as [5–8].

In this paper, we use a motion estimation algorithm based on the optical flow approach. While we do not claim that this method is optimal for any particular task, optical flow methods are present in many state of the art algorithms for motion estimation and biomedical imaging. The particular optical flow algorithm applied here, described in Sections 3.2 and 3.3, has the additional advantage of combining several generic image analysis operations (convolutions, interpolations, and iterative solution of partial differential equations). This makes it a good exemplar case to illustrate the possibilities and limitations of biomedical image analysis using GPUs, which is one of the aims of this paper.

### 3.2. Hybrid Motion Estimation Algorithm

In this paper, we use the motion estimation approach proposed by Bruhn et al. [9], which combines classic solutions to optical flow estimation proposed by Horn and Schunck [10] and Lucas and Kanade [11], as the baseline version. There are several reasons why we believe this is a particularly relevant algorithm for our purpose. In [9], Bruhn et al. reported excellent results including a quantitative comparison in which the algorithm is shown to outperform a number of previously published optical flow approaches. In their subsequent paper [12], they proposed different means of improving the computational performance of the algorithm in a uniprocessor platform, which makes it an excellent example to explore the peculiarities of multicore versus single-core implementations. Finally, the algorithm contains a number of individual operations which are commonly found in medical image analysis applications, and thus could be reused.

Optical flow methods are based on the assumption that corresponding points in two consecutive frames of an image sequence have the same intensity values. This condition can be linearized considering only the first terms of the Taylor expansion, which in the case of 3D images gives

(1) $$I_{x}u+I_{y}v+I_{z}w+I_{t}=0,$$

where *I*_*x*,*y*,*z*,*t*_ are spatiotemporal partial derivatives of the image pixel intensities *I* and *u*,*v*, and *w* are displacement vector components. Equation (1) is a constraint equation and direct estimation of *u*, *v*, and *w* by minimising the derivatives therein is an underdetermined problem, and additional constraint(s) are required. Under this circumstance, the most that can be done is obtain the projection of the vectors in the corresponding direction of the image gradients *I*_*x*,*y*,*z*_, which is referred to as the aperture problem.

Several alternatives have been proposed to solve the aperture problem. In [10], Horn and Schunck propose a variational approach, where it is assumed that the motion field is smooth in the neighbourhood of estimation and it seeks to minimize

(2) $$\begin{array}{cc} E\left(u,v,w\right) & =\iint ({\left(I_{x}u+I_{y}v+I_{z}w+I_{t}\right)}^{2}\\ & \;\;\;\;+\alpha \left({\left|\nabla u\right|}^{2}+{\left|\nabla v\right|}^{2}+{\left|\nabla w\right|}^{2}\right))dx\;dy\;dz. \end{array}$$

In other words, (1) is transformed into a cost term (*I*_*x*_*u* + *I*_*y*_*v* + *I*_*z*_*w* + *I*_*t*_)^2^ to be minimized along with a regularization term *α*(|∇*u*|^2^+|∇*v*|^2^+|∇*w*|^2^) which assures well-posedness. Furthermore, *α* is the weight of the regularization term which links intensity variation and motion.

In [11], Lucas and Kanade assume that the local motion is constant within a certain neighbourhood *ρ*; this provides a system of linear equations which can be directly solved. The method adopted in this paper, originally presented by Bruhn et al. [9], utilises a hybrid of both the approaches described above. This approach exploits regularization both at the local [11] and at the global level [10]. In short, the approach involves calculating the matrix

(3) $$J_{\rho }\left(\nabla_{4}I\right)=K_{\rho }*\left(\nabla_{4}I\nabla_{4}I^{T}\right),$$

where, following the notations from Bruhn et al. [9], ∇_4_*I* is a column vector containing the derivatives of *I* with respect to *x*, *y*, *z*, and *t* and *K*_*ρ*_ is a Gaussian kernel with variance *ρ*, which is convolved with each of the matrix components. *J*_0_ is used to represent the matrix before the application of the Gaussian filter. This leads to following functional to be minimized:

(4) $$\overset{}{\underset{\Omega }{\int }}\left(w^{T}J_{\rho }\left(\nabla_{4}I\right)w+\alpha {\left|\nabla w\right|}^{2}\;\right)dx\;dy\;dz,\;$$

along with the definitions of

(5) $$w={\left[u,v,w,1\right]}^{T},\;\;{\left|\nabla w\right|}^{2}={\left|\nabla u\right|}^{2}+{\left|\nabla v\right|}^{2}+{\left|\nabla w\right|}^{2}.$$

The functional in (4) is minimized by solving its corresponding Euler-Lagrange equations

(6) $$\begin{array}{c} 0=\Delta u-\frac{1}{\alpha }\left(J_{11}u+J_{12}v+J_{13}w+J_{14}\right),\\ 0=\Delta v-\frac{1}{\alpha }\left(J_{21}u+J_{22}v+J_{23}w+J_{24}\right),\\ 0=\Delta w-\frac{1}{\alpha }\left(J_{31}u+J_{32}v+J_{33}w+J_{34}\right), \end{array}$$

where Δ*u* represents the Laplacian of *u*, and we use, as in [9], the notation *J*_*ij*_ to refer to the values at position (*i*, *j*) in the matrix *J*_*ρ*_(∇_4_*I*). With this, (6) can be expressed as a system of linear equations in the form of *Ax* = *b*, where

(7) $$A=\nabla_{4}I\times \nabla_{4}I^{T},\;\;x=\left[\begin{array}{c} u\\ v\\ w \end{array}\right],\;\;b=\left[\begin{array}{c} \Delta u\\ \Delta v\\ \Delta w \end{array}\right],$$

where the Laplacian for a spatial point *i* can be approximated from the neighbourhood elements as below:

(8) $$\Delta u=6u_{i}-\underset{j\in N(i)}{\sum }\frac{u_{j}}{h^{2}}.$$

We represent the three-dimensional six-neighbourhood of *i*, as *N*(*i*) and *h* is the image resolution.

The total number of equations/unknowns given in (6) is 3*N*_*x*_*N*_*y*_*N*_*z*_, which means an iterative solving method needs to be used. In [9], Bruhn et al. used the successive over-relaxation (SOR) method. The SOR method changes the motion values on the fly; that is, the calculation of motion at iteration *k* will use the motion values already calculated at that iteration. An alternative is the Jacobi method, which bases the calculation of all motion values at iteration *k* only on the values from the previous iteration, thus allowing a more efficient parallelization. The equation to calculate *u*_*i*_ for each voxel *i* is

(9) $$u_{i}^{\left(k+1\right)}=\frac{\sum_{j\in N(i)}^{}u_{j}^{\left(k\right)}-\left(h^{2}/\alpha \right)\left(J_{12,i}v_{i}^{\left(k\right)}+J_{13,i}w_{i}^{\left(k\right)}+J_{14,i}\right)}{\left|N\right|+\left(h^{2}/\alpha \right)J_{11,i}},$$

where the superscript *k* denotes the iteration number and |*N*| is the number of neighbours of voxel *i* within the domain. *J*_12,*i*_ represents the value of the component (1,2) of the *J* matrix in (3), calculated at voxel *i* in the image and calculated at the start of the iterative procedure (i.e., independent of iteration number *k*). Similar expressions can be easily found for the components of the motion field along the *y* and *z* axes, respectively, *v* and *w*. The algorithm thus starts with an initialization for *u*, *v*, and *w* (zero in our case) and a precalculation of the *J* values, and iteratively calculates the values of *u*^*k*^, *v*^*k*^, and *w*^*k*^ until convergence is reached. Calculation of *u* at voxel *i*, thus, requires the values of *u*, *v*, and *w* at the same voxel *i* and the values of *u* at neighbouring voxels *j*, all from the result of the previous iteration.

Using a linear approximation as in (1) works only in the case of very small motions, which is overly restrictive for general medical imaging applications. In order to overcome this limitation, it is possible to apply the whole procedure within an iterative framework, where the motion field is calculated, applied to the moving image, and the motion estimation process starts again using this newly resampled image. Note that this does not require recalculation of the spatial gradients *I*_*x*_, *I*_*y*_, *I*_*z*_, as these are calculated on the fixed image. In the same way, the whole procedure can be embedded in a multiresolution framework without any major changes. We present the overall algorithm in Algorithm 1 and discuss in detail below. 

 In summary, the algorithm can be divided into these sub-tasks, whose GPU implementation is described below.

1. Calculate the derivatives of the image intensities with respect to spatial and temporal coordinates: *I*_*x*_, *I*_*y*_, *I*_*z*_, and  *I*_*t*_.
2. Calculate the cross-products of the derivatives (this would correspond to the matrix *J*_0_(∇_4_*I*)).
3. Convolve each one of the components of the matrix above with a Gaussian filter *K*_*ρ*_ to produce *J*_*ρ*_(∇_4_*I*).
4. The resulting system of linear equations given in (6) are solved using * eqnSolve* which deploys an iterative technique. This necessitates estimating the Laplacian values using * Jacobi*.
5. Apply this motion field to all the frames of a moving image along with resampling wherever necessary.
6. All of the above are applied repeated *R* times where the solution converges.

To simplify the notation, in Algorithm 1 and in subsequent sections, we assume that the motion vectors are calculated between two images *I*_1_ and *I*_2_, corresponding to two consecutive frames in the temporal sequence.

### 3.3. Enhanced Volume-Conserving Motion Estimation

Cardiac muscle is to a large extent incompressible [13, 14], and thus, in this application, it is important for the estimated motion field to preserve the original volume locally. A number of algorithms have been proposed to estimate incompressible motion fields, with the Jacobian being commonly used as a measure of volume change. In this paper, we use the variational optical flow first introduced by Song and Leahy [15], where an additional term is introduced in the minimization to favour divergence-free motion fields, which together with the diffusion-free term ensures volume preservation. Equation (4) thus becomes

(10) $$\int_{\Omega }\left(w^{T}J_{\rho }\left(\nabla_{4}I\right)w+\alpha {\left|\nabla w\right|}^{2}+\beta \cdot \mathrm{div}\;\left(w\right)\right)dx\;dy\;dz.$$

The solution is then computed using the Euler-Lagrange equation, similarly to the derivation presented above. The original algorithm presented in Algorithm 1 can be modified to account the preserving term we introduce here.

## 4. GPU Parallelization

The original algorithm exhibits abundance amount of parallelism at the pixel level. The CUDA architecture, where parallelism exists at the single instruction multiple data (SIMD) level, is particularly suitable for exploiting such a fine-grained parallelism. Although exploiting this appears rather trivial at the algorithmic level, the data placement and management posed considerable challenges in realising the algorithm. Furthermore, the continuous evolving of the architecture has a direct impact on the way the algorithms are realized.

The raw-data for the computation is represented as a vector of *N*_*x*_*N*_*y*_*N*_*z*_ elements, with the best possible spatial locality along one of the dimensions (in our case, this is *x*). In the case where the size of the raw image to provide any undesirable effects for coalesced access, we pad the image appropriately. This leads to constant strided access along other two dimensions (stride of *N*_*x*_ along the *y*-axis and *N*_*x*_*N*_*y*_ along *z*-axis). Although nonlinear layouts may provide some performance benefits, we have not considered them, to minimize the addressing issues. Initial set of images will be denoted by *I*_1_ (fixed image) and *I*_2_ (moving image).

As we have discussed in Section 2, the complexity of modern GPU architectures in terms of data placement and management directly impacts the way that the algorithm is realized. In particular, the Fermi architecture supports both shared and cache memory. Though predetermined, a finite pool of memory can be used as a full shared memory, or as cache memory or in hybrid fashion. There is no well determined method for establishing which configuration will lead to better results. In our case, the repeated application of the algorithm may benefit from shared-memory, but this brings additional overheads to the data movement. Alternatively, the shared-memory functionality can be turned off, and we could configure the available memory as a level-1 (L1) cache, which will simplify the management. We foresee that since the accesses are constant strides, the latter configuration is likely to provide better results. However, to verify this, we implemented both methods. In the following sections, we outline how we have implemented this among a set of key functions which are central to the motion estimation algorithms outlined in Section 3.

### 4.1. Gradient Calculation

From the two initial three-dimensional images (*I*_1_ and *I*_2_), the gradient values are calculated using a forward difference approximation: *I*_*x*_(*x*, *y*, *z*) = *I*(*x* + 1, *y*, *z*) − *I*(*x*, *y*, *z*), assuming that two consecutive voxels are separated by unit distance. These are calculated on the fixed image *I*_1_. In the same way, the value of the temporal derivative is approximated by the finite difference *I*_2_(*x*, *y*, *z*) − *I*_1_(*x*, *y*, *z*). Border voxels are dealt with by assigning their corresponding derivatives to zero, rather than assigning periodic boundary conditions. The cross-products are then calculated, producing a total of nine extra values per voxel: *I*_*x*_^2^, *I*_*y*_^2^, *I*_*z*_^2^*I*_*x*_*I*_*y*_, *I*_*x*_*I*_*z*_, *I*_*y*_*I*_*z*_, *I*_*x*_*I*_*t*_, *I*_*y*_*I*_*t*_, and *I*_*z*_*I*_*t*_.

**Figure 3:**
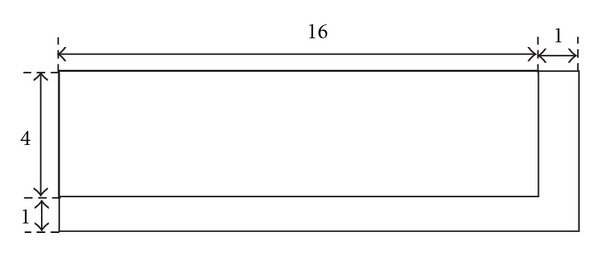
An example of the shared memory tiles used complete with a halo on two sides to store the extra values that are required.

**Figure 4:**
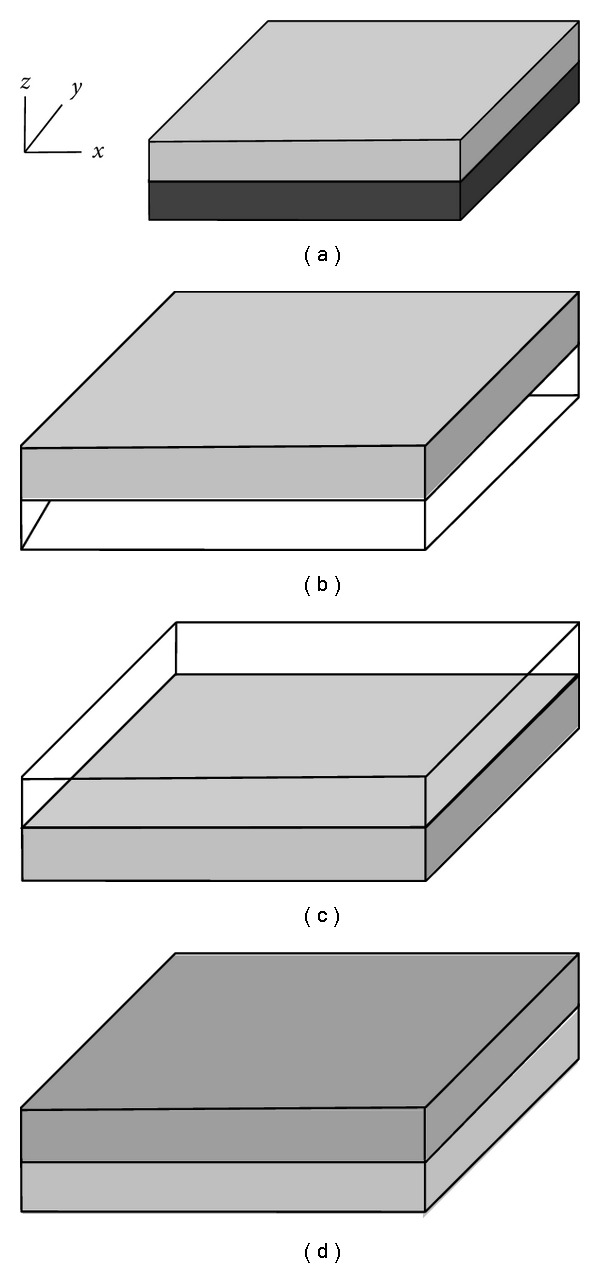
(a) the initial state of the shared memory. (b) after the plane *z* has been deleted. (c) the top tile (*z* = 1) has been moved into the position of the bottom tile (*z*). (d) new data with the next *z* coordinate value is then loaded into the space created by moving the top tile. In each instance, the different shades of Gray represent different layers within the piece of data that we are reading from into shared memory.

The simplest strategy to parallelize this calculation would be to allocate a thread to every pixel. However, given the memory arrangement described above, this would result in each thread performing five loads from main memory (the values of *I*_1_ at the corresponding voxel, the three forward neighbours in *x*, *y*, *z* and the value of *I*_2_), including one that is uncoalesced (the value *I*_1_(*i* + 1, *j*, *k*) as *x* values are consecutive, and so they do not have an appropriate stride for coalesced memory access). To reduce this overhead, we could either rely on the L1 cache or use the shared memory, a common technique we will be reusing in realising other key routines. The data is first partitioned into cubes along the *x* and *y* directions (each cube containing the full range of *z* values). As the computation for each cube is looking forward, for each cube, we also require a halo of size one along the *x* and *y* directions, as shown in Figure 3. However, the partitioned cube may still not fit the shared memory. For this reason, we process the image as slices along the *z* direction. This introduces a halo of size one along the *z* direction as well. This means that the shared memory for each block of threads needs to be of size (*X* + 1, *Y* + 1,2). Image values are then loaded into shared memory, with each thread loading its interior (i.e. nonhalo) voxel in its image *I*_1_ at that *z* value, and the value of the corresponding voxel in image *I*_2_. The halo values are then loaded in by a subset of the threads. This subset is constructed by giving each thread a number *k* such that *k* = thread  *Idx*.*x* + thread  *Idx*.*y* × *X* and then selecting the threads where *k* < *X* + *Y* + 1. Each of these threads will load one value for the halo in the *x* and *y* directions. This means each thread needs to perform a maximum of three loads instead of five, with *Y* + 1 of these being uncoalesced per *z* value. As mentioned before, loading all the values in the *z* direction in one go is not possible with current shared memory sizes of only 64 KB per group of eight processors on these cards. Thus, we only store the values for *z* and *z* + 1 in the shared memory at any one time. However, the number of loads from global memory is kept down by moving the data around within the shared memory as the *z* values change. Once all the computations have been performed for the lower *z* plane in the image, this plane is discarded from the shared memory. Then, the *z* + 1 plane is copied allowing it to become the new *z* plane. Once this has been completed, a new *z* + 1 plane is loaded. Diagrams demonstrating this can be seen in Figure 4. For this application, we cut the data into pieces of size 32 × 4. This size was chosen through experimentation and maintaining the necessary constraint that the *x* dimension is a multiple of 16 in order to maintain coalesced memory accesses.

### 4.2. Smoothing

 After the cross-products of the derivatives have been calculated, a Gaussian filter is applied to each of them. This is performed by convolving the image with a kernel approximating the Gaussian, in the following way:

(11) $$f_{\sigma }\left(x,y,z\right)=\overset{i=K_{x}}{\underset{i=-K_{x}}{\sum }}\overset{j=K_{y}}{\underset{j=-K_{y}}{\sum }}\overset{k=K_{z}}{\underset{k=-K_{z}}{\sum }}g_{\sigma }\left(i,j,k\right)f\left(x-i,y-j,z-k\right),$$

with *g*_*σ*_ being the values of the kernel obtained by sampling a Gaussian function. The kernel has a size (2*K*_*x*_ + 1,2*K*_*y*_ + 1,2*K*_*z*_ + 1), where the values of *K*_*x*_, *K*_*y*_, and *K*_*z*_ are captured as *K*_*ρ*_ in Algorithm 1; the values of *K*_*x*_, *K*_*y*_, and *K*_*z*_ need to be large enough to provide an accurate approximation to the Gaussian but not too large to avoid unnecessary calculations. Large values of sigma require large kernels, and thus impose a big computational load. As an alternative, the 3D convolution can be separated into three one-dimensional convolutions, one in each of the *x*, *y*, and *z* directions. This is the approach we have used. For simplicity of notation, in the next Section we assume *N*_*x*_ = *N*_*y*_ = *N*_*z*_, and we use the value *K* = 2*N*_*x*_ + 1

As mentioned above, a naive implementation would just use a separate thread for each value within a given set. However, this approach also suffers from the number of global memory loads, which this time are a function of the kernel size (*K*) being applied, giving 3*K* loads from the dataset plus 3*K* accesses to load the kernel for the convolution in each direction. Of these, 3*K* accesses to load the kernel, and the *K* accesses to perform the convolution in the *x* direction will be uncoalesced. Clearly, this will cause a server bottleneck, so once again, we use the shared memory to reduce the cost of this. Additionally, we also use the constant memory on this occasion.

Half of the global memory accesses can be removed by simply storing the kernel in constant memory instead of global memory. This change does require a maximum size for the kernel to be set, but as this maximum can be in the thousands, this is not a restriction on the design.

The method for utilising the shared memory is the same as in Gradient calculation. Each thread loads some of the data ensuring coalesced memory accesses, and then all the threads share this loaded data to perform the computation. However, as the computation takes the form of three separate passes due to the separation of the three-dimensional convolution into three separate one-dimensional linear ones, it has to be formed from three separate CUDA invocations. This means that in any given invocation, the computation will only be looking in one direction. This is important, as the shared memory is not large enough to store sufficient information to look in all three directions at the same time. This behaviour also means it is necessary to construct a different style of solution for the *x* direction to the *y* and *z* directions to take into account the need for coalesced memory accesses.

### 4.3. Solving the System of Linear Equations

For solving the system of linear equations, we implemented the two different variants of the Jacobi method: one version will rely on the L1-cache (without any shared memory), and the other will use the shared memory. Both approaches reduce the number of global memory accesses, in particular uncoalesced ones. In the case of the shared memory version, as before, we partition the data into tiles each with a halo, and these tiles are slices along the *z* dimension. However, on this occasion, given both the forward and the backward neighbours are used, the halo occupies both edges in both *x* and *y* as shown in Figure 4, and we maintain three tiles at any given moment bar the first and last values in the *z* direction, as zero padding at the borders is assumed. Due to the limited size of the shared memory, each of the *z* planes is computed in turn, so increasing the available memory for a given computation and so increasing the ratio of values computed to halo values loaded.

### 4.4. Intensity Interpolation

As explained above, due to the linear approximation introduced in (1) the above process has to be repeated in an iterative motion estimation/interpolation cycle. Having estimated the motion between the two images, it is now necessary to apply this motion to the moving one and interpolate the image intensities at the new positions. We use a trilinear approach, where the value of the image at each position is linearly interpolated from the values of their eight immediate neighbours. While there is a range of interpolation techniques including nearest neighbour, tricubic and different spline-based methods, in this application the trilinear method was deemed a good compromise between accuracy and speed. However, staying in line with the over arching aim of this work, we ensured that our framework is easily amenable to a different method.

The smoothness constraint in (4) and (10) means that there should be a high degree of locality associated with the reads required to sample for pixels that share locality in the original image. Despite this pattern, there is no way to determine the locations in advance, so it is not possible to use the shared memory to save on access times or to overcome the inevitable uncoalesced memory accesses. For these reasons, we turned to the texture memory to provide caching for the data accesses to improve the performance of this phase. Once the original image is mapped to a texture, it would have been possible to get the texture functionality already available in the device to perform the interpolation instead of writing new code. However, this is only implemented to a sufficient accuracy for displaying pixels on computer screens, to around four decimal places, and is inflexible in that we would be restricted to the interpolation techniques supported by the cards, rather than being able to extend this code to perform alternative interpolation techniques. As such, we map the data structure to a texture with a call from the host and then perform the interpolation on a one thread per voxel basis. Each thread calculates the location of and extracts the eight closest voxels from the texture memory. Having done this, it performs the calculation and saves the result back to the main memory. Because of the use of the texture memory as a cache, the locality of the eight pixels, and the locality of any other interpolated points within the blocks executed on a given group of cores results in both coalesced memory accesses and data reuse.

The use of the texture memory instead of the shared memory to overcome the limitations of the memory system makes this piece of code by far the simplest and demonstrates how much clearer CUDA code can be once all concerns about memory management are abstracted away. However, experiments with the use of texture memory shows that it is actually two orders slower than shared-memory counterpart, and thus, we will not be discussing this any further. We attribute the overheads to the losses. 

## 5. Experimental Evaluation

### 5.1. Experimental Procedure

 The task of performance comparison of an application on CPU- and GPU-based systems is highly dependent on a number of factors. These include the underlying operating system, compilers used, optimization flags, order of the optimizations and caching policies of the platforms in question [16]. With this in light, it is difficult to conclude that an application will always lead to performance improvement on another platform. For this reason, to gain more insight into the benefits, we use three different systems to compare and analyse the performance results. The details of the systems on which we performed our experiments are shown below in Table 1.

There are different metrics by which we could compare the benefits. In this paper, we treat the version compiled for the host CPU as the baseline version. The computation on the GPU involves more than raw-computation on the GPU cores. This includes data transfers and associated managements. However, in our context, we see that the data will persist in the GPU for subsequent runs, and therefore, we report the performance results excluding the data transfer times. For the rest of the section, we evaluated the performance of the algorithm as follows.

1. For each system, we perform the runs a number of times in an unperturbed condition, and we chose the median of the measurements.
2. We use a database of three-dimensional image sequences of varying sizes to test our algorithm under different configurations (see below). The database includes synthetic images wherever needed, which does not affect the results.
3. The nonsystem-specific and algorithm-specific parameters are tested for their influence on the overall performance of the algorithm. This includes the kernel size and number of iterations.
4. The overall motion estimation algorithm, as discussed in the previous sections, contains a number of components and each of them gain significant speedups when run on the GPU. We evaluate the speedups gained by different components.
5. Different variants of the implementations are used to assess the impact of shared memory, L1 and L2 cache memories on the algorithm. For this, we run the following variants.
  - A shared memory version. This is available on all systems. In the modern systems, the L1-cache is turned off and the full pool of memory is used as shared memory. On the C1060 system, this is the standard configuration.
  - A nonshared memory version. On Fermi-based systems (GTX480 and C2070), this effectively turns on the L1-cache. In addition to this, this approach simplifies the overall programming as complicated techniques such as tiling are not needed, and thus purely relying on the loading resolution of the cache controllers on the GPU.
  - A no-L1 mode. This turns off the L1 and shared memory mode and thus purely relies on L2-cache. This configuration does not exist on the older systems (C1060).

### 5.2. Experimental Results

**Figure 5:**
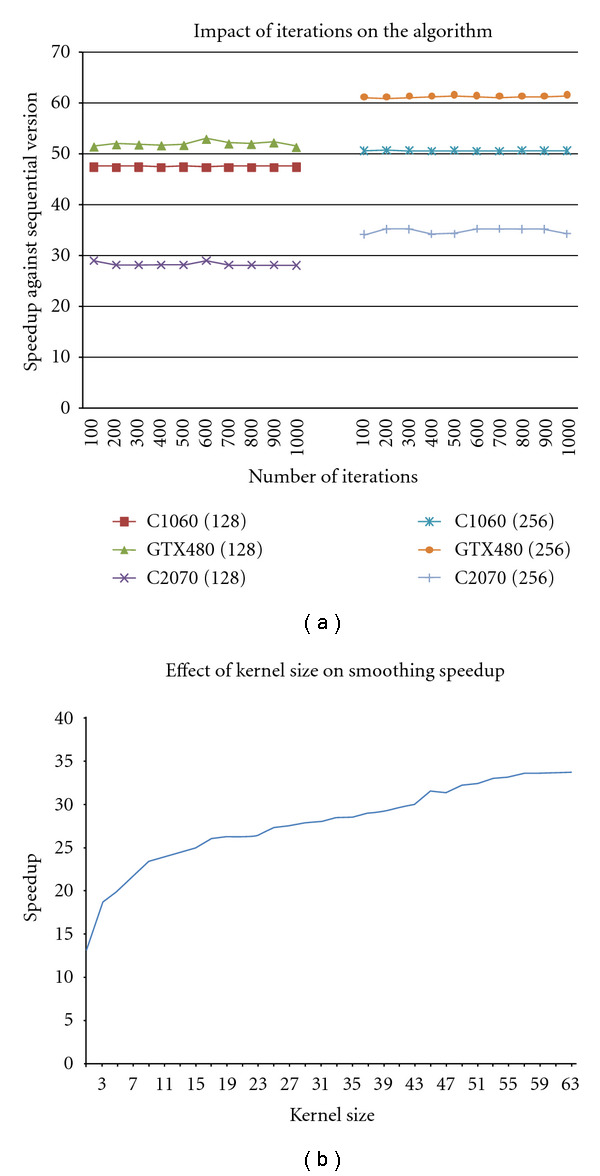
(a) shows the impact of number of iterations in the overall speed up of the algorithm. We show the impact for two different image sizes. (b) shows the impact of kernel size on the overall speed up of the algorithm.

We first present the impact of the number of iterations (denoted by *R* in Algorithm 1) and of the kernel size (denoted by *K*_*ρ*_ in Alogrithm 1), on the overall speedup in Figure 5. We evaluate the impact of the number of iterations using two different fixed size images (128^3^ and 256^3^ images) on all three platforms, for a range of iterations. For each platform, we pick the best performing versions (among shared memory, nonshared memory and non-L1-mode) and then we vary the number of iterations. As observed, the number of iterations does not have a noticeable impact on the overall speedups across all platforms. Although varying the image size changes the maximum speedup (speedup increases as image size increases), for a given image, the number of iterations do not alter the speedup by a large degree. This is because, although increasing the number of iterations benefits from overall reuse per transfer, the inter- and intraiteration spatial locality in the cache is not in favour of the application. This essentially carries an important message: although the execution time for increased number of iterations will rise, the speed up will not be affected. However, the kernel size has an impact on the overall performance of the algorithm. As the kernel size increases, the number of accesses to memory per floating point operation (FLOP) decreases and this improves the GPU speedups. Furthermore, if the kernel size were to exceed the size of the CPU cache, this observation will change considerably.

Provided that for a given case, the number of iterations and the kernel sizes are fixed, the overall speedup is only affected by the size of the image. In the remaining part of this section, we keep the number of iterations and the kernel size constant, and we only vary the image size.

**Figure 6:**
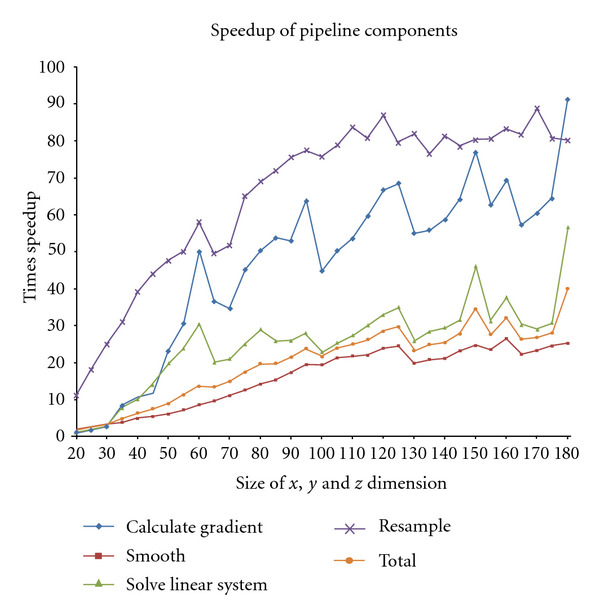
Speed up of the different components of the motion estimation algorithm for different size of images on the C1060 platform.

As stated above, the overall motion estimation algorithm has a number of operations, which are componentized in our case, and their speedups on the GPU vary with the problem size. This is shown in Figure 6 for the C1060 platform. Here, all components of the algorithm show significant gains in speedup. The kernel size for the smoothing is 15. Different parts of the pipeline have different access patterns, leading to different overall behaviour. However, both the gradient calculation and linear system solution use the same access patterns both on the host and the GPU, explaining the similar shape. The presence of local * spikes* in speed up is due to the different optimal sizes between CPU and GPU. In our case, we observe that the Gaussian filter achieved a speed up of around 25 times, and the data generation and image interpolation achieved speedups close to 90 times in the best case. The worst case values (slowdowns) were observed for very small image sizes (e.g., around 5 times slowdown for Gaussian), which are not shown here. However, the overall speed up is much less than the best speedups of all components and this is currently limited to 60 times. We also observed the highest percentage of the time being spent solving the linear equations and smoothing the data.

Having presented the performance of fine-grained aspects of the GPU performance, we present the overall performance behaviour of the algorithm on different systems. As stated in the previous section, different variants of the implementations are tested for their performance.

**Figure 7:**
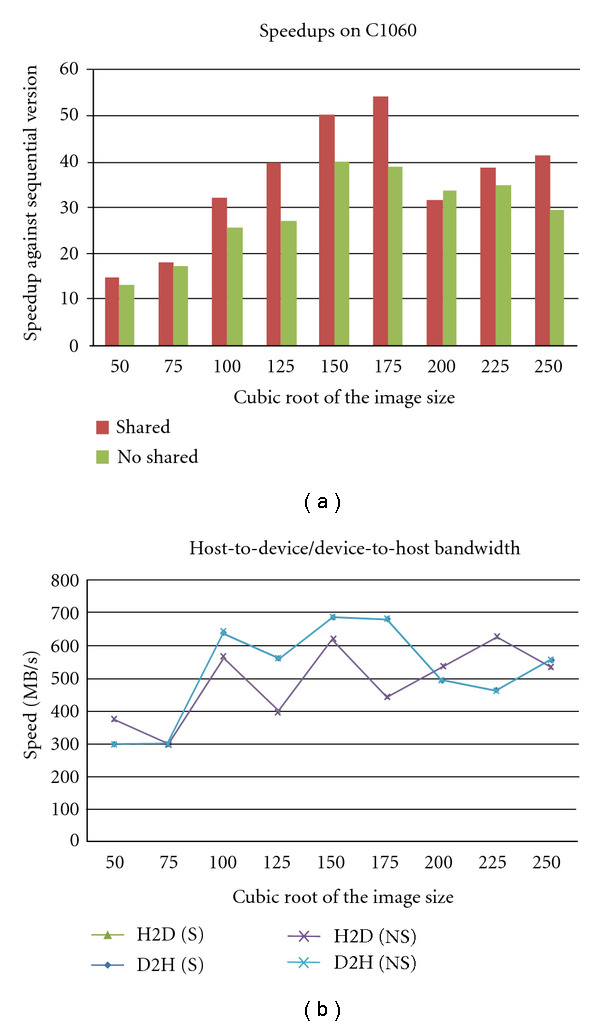
(a) shows the overall speed up of the algorithm on the C2070 system with the data transfer times excluded while (b) shows the variation of transfer speed with the image size.

The speedup on the C1060 system is shown in Figure 7 for two different configurations. One with the shared memory being exploited and the other one without any shared memory utilization. In overall, the shared memory implementation is faster than nonshared memory implementation. For the reasons discussed in Section 2, accessing shared memory is faster than accessing the device memory. This improved the implementation so less time is spent on wait states, thus the overall speedup gains. One other observation, for which further investigations are needed, is sudden slowdowns at image size of 200^3^ which are more pronounced on the shared memory implementation.

Reporting speedups as in the figures above can be sometimes slightly misleading. In particular, this is true when slowdowns on the CPU side are translated as speedups. For this reason, we present the raw runtimes in Table 2. In addition to this, the transfer speed rate between the host and device and vice versa varies with data size. As per the configuration, the rate is biased towards the direction in which the transfer occurs (data transfer rate from device to host is much higher than transfer rates from host to device) but rarely achieves the peak rate of respective systems.

**Figure 8:**
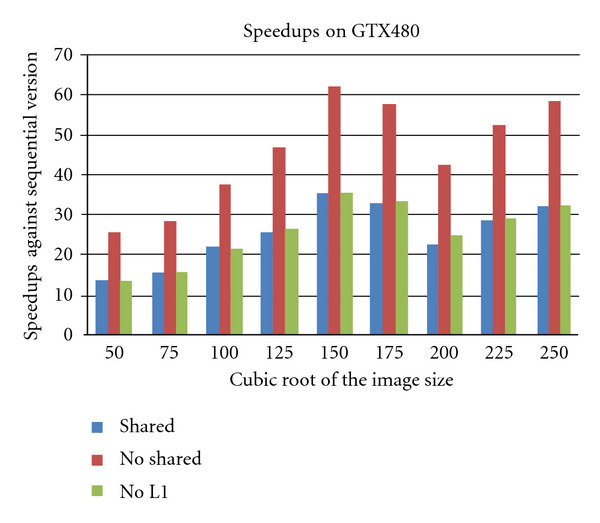
The overall speedup of the algorithm on the GTX480 system for different image sizes with the image size (with the data transfer times excluded).

Figure 8 shows the performance of the algorithm on the GTX480 system. Since GTX480 GPU is based on the Fermi architecture, the GPU contains two levels of caches, (L1 and L2) one which can be configured either as a shared memory, as a L1-cache or a mix of both. We ran our implementation under three different configurations. First, we ran the algorithm with no-shared memory option. This is the default and triggers the usage of L1 and L2 caches of the system. Under this setup, the implementation does not need to have any extensive shared-memory mapping procedures. Then, we performed the same experiment with the shared memory variant of the implementation. Under this configuration, the L1-cache is fully configured as a shared memory system and our implementation takes the full responsibility of the loading and data placement. Finally, we run the implementation with the L1-cache being completely turned off. This renders both the shared memory and L1 not available to the application, thus fully relying on the L2 cache, which cannot be turned off.

As can be observed in Figure 8, the default configuration outperforms the other two versions. In other words, extensively tuning the application for shared memory usage is not optimal under the new architecture. Two reasons can be attributed to this failure. Firstly, the data management and placement overheads cannot compete with the automatic placement provided by the L1-cache. Secondly, the L2-cache deploys the inclusive policy, which feeds the L1 without any extra wait states.

However, turning off the L1-cache does not affect the performance significantly since the L2-cache is active. Very close inspection of Figure 8 and runtimes in Table 3 reveal that even after turning off the L1-cache, the overall performance of the shared memory implementation is outperformed. Again, we explain this based on the efficiency and loading policies of the cache controllers on GPU. Furthermore, a performance drop at the image size 175^3^ is observed similar to what was observed on the C1060 system, but here, the nonshared memory implementation suffers from slowdowns.

**Figure 9:**
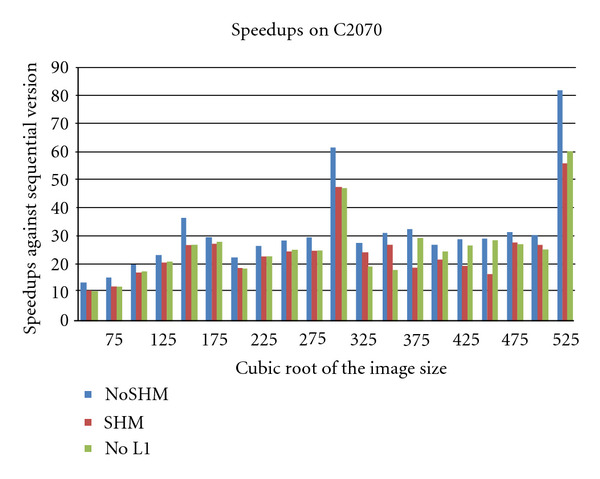
The overall speed up of the algorithm on the C2070 system for different image sizes (with the data transfer times excluded).

Finally, we present the performance of the algorithm on the C2070 system in Figure 9. Since the system has no device memory, we were able to run the algorithm with considerably large image sizes (up to 525^3^).

Similar to the GTX480 system, the default configuration, where no shared memory was utilized, performs better than the other two version. All the other observations align with the previous observations on GTX480 system. Furthermore, as before, even relying on the Level-2 cache is sufficient enough to gain substantial speedups. Furthermore, a performance drop observed on the C1060 and GTX480 systems observed here as well, where the observation is repeated. Although all of these observations have been visible in other systems, one subtle feature of this C2070 system is that it has a built-in error checking and correction (ECC) mechanism for the GPU memory.

**Figure 10:**
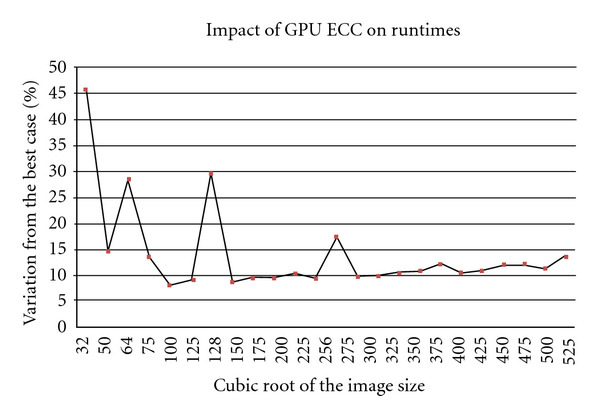
The variation of the runtime when ECC is enabled as a percentage of the best case for the C2070 system.

Historically, in the context of GPUs, error rates were not an issue—as this only affected the color of the pixel. However, this is no longer the case with GPUs being used as part of the HPC systems as the soft error rates in DRAM are high. Despite this, previous generations of GPUs did not support ECC and only recently this support has started to arrive. In our case, C2070 supports ECC. However, the ECC comes with the penalty on runtimes due to overheads associated with a large number of check bits. To evaluate the impact of ECC, we ran the algorithm with and without ECC. To avoid any direct influence of L1 on this, we turned off the level-1 cache. We report the variation of the runtimes as a percentage of the best case, the non-ECC version, in Figure 10. However, in our case, we did not see any difference in accumulated errors, which is a random event. As can be observed, the variation diminishes as image sizes increases but is more pronounced at the power-of-two problem sizes. At the power-of-two problem sizes, the number of check bits required for ECC is higher than for nonpower-of-two problem sizes [17], and thus overheads are considerably high. We assume that with increasing data size, the accesses are more consolidated to blocks, and thus the number of separate checks reduces.

## 6. Conclusions

In this paper, we investigated the mapping of an enhanced motion estimation algorithm to a number of GPU-specific architectures, resulting challenges and benefits therein. Using a database of three-dimensional image sequences, we showed that the mapping leads to substantial performance gains, up to a factor of 60, and can provide near-real-time experience. By doing this, we gained more insight into the process. From our investigation, we observed the following.

- Although the presence of different memory subsystems are key in GPU programming, their significance is diminishing. We witnessed this simply with the use of shared and constant memories against level-1 and level-2 caches. Partly, this observation is very influential across image processing applications—where working with large amounts of data is a fundamental requirement. In modern Fermi-based systems, the loading resolution of the cache controllers amortizes the overheads in managing different memory subsystems.

(ii) In three-dimensional image processing applications, the spatial locality can only be exploited along one dimension in the CPU, while there is no spatial locality in the other two dimensions. This leads to benefits along one of the dimensions on the CPU. Meanwhile, on the GPU-front, nonspatially local dimensions benefit from coalesced memory access. However, memory accesses along the remaining dimension do not benefit from coalesced memory access. These two facts are difficult to assess without detailed profiling but are evident in the fact that coalesced access leads to better performance.

(iii) In three-dimensional image processing applications, increasing the number of smoothing iterations on the GPU will not change the overall speedup although it increases the absolute runtimes.

(iv) Even in the absence of the level-1 (L1) cache, the performance was sustained by the level-2 cache.

(v) Error correction and checking (ECC) is necessary for reliable outputs. However, wherever possible, this can be traded off for performance.

(vi) In a typical image processing application, the motion estimation pipeline is repeatedly applied for subsequent frames of image sequences, and therefore, it is valid to assume that the data will persist on the device and long-term runtime benefits will amortize the cost of host-to-device and device-to-host transfers.

(vii) The overall cost per performance is very attractive. The cost of a 512-core GPU is only a quarter of a 16-core CPU-based system (as of mid 2011). Nevertheless, the GPU-based system yields noticeable performance benefits. Furthermore, the evolving and simplified architectural and programming models, makes this an excellent option for biomedical image processing applications.

Although our work has exploited several different aspects of the GPU-architecture, there are several different aspects which may be improved.

- For extremely large datasets, where the device memory cannot hold the entire dataset, it may be necessary to perform distributed processing using multiple GPUs. Although both our algorithm and the software framework can easily be extended to cover this, the immediate benefits on near-real-time experience is not known.

(ii) The current Fermi-based GPUs support concurrent kernel execution, which permits launching multiple kernels in a concurrent fashion. This feature essentially liberates the GPU and unlocks it from traditional SIMD-style processing. The exact benefits still need to be investigate especially in the context of three-dimensional image processing applications.

(iii) To alleviate the intricacies relating to conditionals, the current version of the algorithm does a fixed number of iterations. A more suitable method is needed to adaptively control the convergence rate.

With all these, we find that although exploiting architectural peculiarities rendered tangible benefits, these benefits are narrowing with the evolving architecture and simplified programming models. The future of GPU architectures will incorporate prefetching [18] and will support abstractions at the higher level.

## Figures and Tables

**Algorithm 1:**
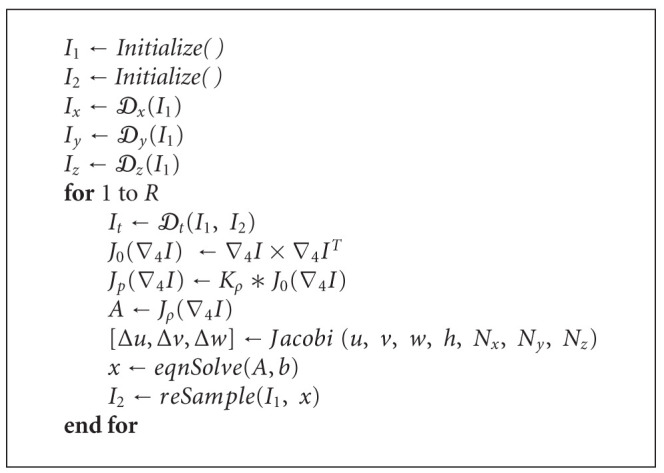
The estimation algorithm without the volume preserving term (see Section 3.3. The meanings of the symbols remain as in the text. *I*_1_ represents a static image *I*(*x*, *y*, *z*, *t*) and *I*_2_ represents a moving image *I*(*x*, *y*, *z*, *t* + 1). First, the Image *I*_1_ is initialized and partial derivatives are computed. *𝒟* denotes the partial derivative operator. Following this, the algorithm is applied repeatedly *R* times. Each time of the iteration, as a precursor to solve (6), values of Δ*u*, Δ*v* and Δ*w* are computed using Jacobi method. The routine * Jacobi* performs this operation. One this is available, all the unknowns, given by *x*, are solved iteratively using the routine * eqnSolve*, which solves a system of linear equations. Then the solutions are used to resample the image to estimate the moving image *I*_2_.

**Table 1:** Details of systems used for evaluation.

Parameters	System 1	System 2	System 3
System name	C1060	GTX480	C2070
Host CPU	Xeon 5110 (Harpertown)	Intel Core i7	Xeon 5650 (Gulftown)
Host CPU speed	1.6 GHz	2.8 GHz	2.67 GHz
Host OS	Ubuntu 10.10 (64 bit)	Ubuntu 10.10 (32 bit)	Ubuntu 10.10 (64 bit)
Kernel	2.6.35	2.6.31	2.6.35
Host RAM	2 GB	4 GB	24 GB
Host L1-cache	64 KB	64 KB	64 KB
Host L2-cache	4 MB	8 MB	12 MB
GPU series	C1060	GTX480	C2070
Compute capability	1.3	2.0	2.0
Device memory	1 GB	4 GB	6 GB
Multiprocessors	24	16	14
Cores per MP	8	16	32
Total cores	192	512	498
GPU L1-cache	16 KB	64 KB	64 KB
(Shared memory)			
GPU L2-cache	N/A	128 KB	128 KB
CUDA version	4.0	3.3	3.2
Compiler flags	-O3 –arch = sm_13	-O3 –arch = sm_2.0	-O3 –arch = sm_2.0

**Table 2:** Raw runtimes of the algorithm on the C1060 system.

Cubic root of image size	CPU time (ms)	GPU time (with shared memory) (ms)	GPU time (without shared memory) (ms)
50	1272	86	96
75	4457	245	257
100	11362	355	443
125	21112	531	771
150	45101	895	1123
175	62310	1147	1594
200	96703	3036	2856
225	134668	3473	3834
250	176238	4261	6016

**Table 3:** Raw runtimes of the algorithm on the GTX480 system.

Cubic root of image size	CPU time (ms)	GPU time (with shared memory) (ms)	GPU time (without shared memory) (ms)	GPU time (without L1) (ms)
50	671	49	26	49
75	2379	155	83	155
100	5788	272	150	265
125	11338	440	240	435
150	23714	677	373	671
175	31369	955	554	942
200	47914	2037	1101	1999
225	66870	2357	1275	2312
250	91610	2864	1564	2823
